# Assessing quality of medical death certification: Concordance between gold standard diagnosis and underlying cause of death in selected Mexican hospitals

**DOI:** 10.1186/1478-7954-9-38

**Published:** 2011-08-04

**Authors:** Bernardo Hernández, Dolores Ramírez-Villalobos, Minerva Romero, Sara Gómez, Charles Atkinson, Rafael Lozano

**Affiliations:** 1 Center for Population Health Research. National Institute of Public Health, Mexico. Av. Universidad 655. Cuernavaca, Morelos, 62508, Mexico; 2 Institute for Health Metrics and Evaluation, University of Washington, USA. 2301 5th Ave, Suite 600. Seattle, WA 98121, USA

## Abstract

**Background:**

In Mexico, the vital registration system relies on information collected from death certificates to generate official mortality figures. Although the death certificate has high coverage across the country, there is little information regarding its validity. The objective of this study was to assess the concordance between the underlying cause of death in official statistics obtained from death certificates and a gold standard diagnosis of the same deaths derived from medical records of hospitals.

**Methods:**

The study sample consisted of 1,589 deaths that occurred in 34 public hospitals in the Federal District and the state of Morelos, Mexico in 2009. Neonatal, child, and adult cases were selected for causes of death that included infectious diseases, noncommunicable diseases, and injuries. We compared the underlying cause of death, obtained from medical death certificates, against a gold standard diagnosis derived from a review of medical records developed by the Population Health Metrics Research Consortium. We used chance-corrected concordance and accuracy as metrics to evaluate the quality of performance of the death certificate.

**Results:**

Analysis considering only the underlying cause of death resulted in a median chance-corrected concordance between the cause of death in medical death certificates versus the gold standard of 54.3% (95% uncertainty interval [UI]: 52.2, 55.6) for neonates, 38.5% (37.0, 40.0) for children, and 66.5% (65.9, 66.9) for adults. The accuracy resulting from the same analysis was 0.756 (0.747, 0.769) for neonates, 0.683 (0.663, 0.701) for children, and 0.780 (0.774, 0.785) for adults. Median chance-corrected concordance and accuracy increased when considering the mention of any cause of death in the death certificate, not just the underlying cause. Concordance varied substantially depending on cause of death, and accuracy varied depending on the true cause-specific mortality fraction composition.

**Conclusions:**

Although we cannot generalize our conclusions to Mexico as a whole, the results demonstrate important problems with the quality of the main source of information for causes of death used by decision-makers in settings with highly technological vital registration systems. It is necessary to improve death certification procedures, especially in the case of child and neonatal deaths. This requires an important commitment from the health system and health institutions.

## Background

Vital registration (VR) constitutes a key element for planning and evaluation of health systems in all countries. In Mexico, the VR system is managed by the National Institute of Statistics and Geography, Mexico (INEGI), in conjunction with the Ministry of Health (MoH) and the Civil Registry offices. The Mexican VR system relies on information regarding cause of death that is registered annually using data from medical death certificates.

According to international assessments, Mexico's VR system is rated among the best in terms of quality and completeness [1]. Important efforts have been made over the past years to improve the coverage and quality of the mortality registry [2], however, there is still room for improvement. Mexico has a Center for Disease Classification (CEMECE), which was established in 1985 and in 2008 was officially recognized by the Pan American Health Organization/World Health Organization as a Collaborating Centre for the Family of International Classifications [3]. The CEMECE is responsible for monitoring the quality and standardization of the use of the 10^th ^revision of the International Classification of Diseases (ICD-10) in all areas of the health system. Since 2007, INEGI established an automated coding system for underlying cause of death, adopting the Automated Coding of Medical Entities (ACME) system [4] used by the US Centers for Disease Control and Prevention and adapting it to the Mexican context. There are currently 25 countries participating in the International Collaborative Effort on Automating Mortality Statistics [5].

The quality of the information provided in the medical death certificate, however, varies according to the personnel responsible for completing it. In Mexico in 2009, 97% of medical death certificates were completed by physicians and 3% by lay people with authorization of the MoH. These figures vary within the country. For example, 99.9% of medical death certificates are filled out by physicians in urban areas and 93.2% in rural areas. Of those medical death certificates filled out by physicians in the Federal District, only 20% of them are filled out by the physician who treated the deceased. In MoH hospitals in the Federal District, this figure ranges from 8.5% in adults, 17.3% in children, and 28.6% in neonatal deaths [6].

Another way to assess quality of death certification is by using the percentage of ill-defined deaths and examining the percentage of deaths at home. In 2009, Mexico registered around 565,000 deaths, 44.4% of which occurred in health facilities, 47.3% at home, and 8.3% in public areas. Of the 66,062 deaths registered in the Federal District, 62% occurred in health facilities. In addition, while 2.1% of deaths were coded as ill-defined at the national level, this figure was 0.5% for hospitals run by the MoH in the Federal District [6].

Studies in various countries [7-13], including Mexico [14,15], have assessed the validity of death certification by comparing the underlying cause of death in the medical death certificate with other sources that provide additional information regarding cause of death, such as medical records. In general, these studies indicate that the concordance between causes of death from death certificates and those obtained using other sources vary by place and cause of death. Most of these studies have been conducted in developed countries, comparing information on underlying causes of death obtained from death certificates with hospital records. They estimate either kappa coefficients or sensitivity and specificity to assess the validity of death certificates. The information from Latin America comes mainly from studies in Brazil, analyzing the validity and reliability of cause of death registration in specific areas of the country [9,10]. In Mexico, these studies have concentrated on infant deaths [14,15]. To our knowledge, there is no single study in Mexico that has analyzed the reliability of causes of death based on death certificates for a wide range of diseases.

The objective of this study was to assess the concordance between the cause of death obtained from the medical death certificate and a rigorously-defined gold standard diagnosis based on medical records in hospitals in the Federal District and the state of Morelos, Mexico in 2009. We measured the quality of official mortality statistics in a sample of deaths that had occurred in medical units with high quality diagnoses. Gold standard criteria used in this study were developed by the Population Health Metrics Research Consortium (PHMRC) as part of a multisite study to validate verbal autopsy questionnaires in diverse populations [16].

## Methods

### Population and sample

The sample was selected from deaths that occurred in public hospitals in the Federal District and the state of Morelos, Mexico in 2009. Following the protocol of PHMRC, 211 neonatal, 94 child, and 1,284 adult cases were selected to cover a list of causes of death that included infectious diseases, noncommunicable diseases, and injuries. The protocol of the PHMRC considered the selection of cases (100 or 30 depending on cause of death) from three main age groups (neonates, children, and adults). A list of these causes and final number of cases included in the study is described in Additional file 1. This list was used as part of the PHMRC project, although some causes of death were omitted in Mexico due to the lack of deaths from those causes. Deaths were identified in 34 public hospitals (see Additional file 2 for more details). Inclusion criteria for the study were deaths that occurred in the selected hospitals between January and December 2009 with a medical record available at the hospital. The age of each patient at the time of death was obtained from hospital records. Deaths were classified as neonatal deaths (first 27 days of life), child deaths (deaths from 28 days to <12 years) and adult deaths (12 years and up), following the general design of the PHMRC project.

### Gold standard cause of death

This study used the gold standard criteria developed by the PHMRC [16]. These gold standard criteria were developed by a committee of physicians involved in the study and underwent multiple cycles of group review. The gold standard criteria classified deaths into three levels based on the degree to which the information from the medical record provided certainty to classify the death as a given cause: level 1, level 2A, and level 2B. Level 1 diagnoses provide the highest level of diagnostic certainty possible for that condition, consisting of either an appropriate laboratory test or x-ray with positive findings, as well as medically observed and documented illness signs. Level 2A diagnoses are of moderate certainty, consisting of medically observed and documented illness signs. Level 2B was used in place of level 2A if medically observed and documented illness signs were not available but records exist for treatment of a particular condition. Level 1 criteria were used for all gold standard cases, and cases classified as level 2A or 2B were only accepted in situations where it proved impossible to gather sufficient level 1 cases for a particular condition. For the analysis in this paper, we present results pooling levels 1, 2A, and 2B gold standard causes of death.

The following is an example of the gold standard criteria for breast cancer. For a case to be considered gold standard level 1 it had to have either an operative specimen with histological confirmation or a biopsy/fine needle aspiration cytology documented in the medical records. To be considered level 2A it had to have a mammography diagnosis and imaging evidence of metastases in other tissues based on CT scan/MRI/x-rays. In cases where the basis for the initial diagnosis was no longer available, the case could be considered level 2B if there was documented evidence in the medical record of the patient having been under treatment for breast cancer at a recognized cancer hospital or cancer unit.

### Cause of death from official statistics

The process of death certification in Mexico involves several participants and includes the generation of two documents for each death: the medical death certificate and the legal death certificate. The medical death certificate is a compulsory document that allows the relatives of the deceased to obtain the legal death certificate in the corresponding civil registry office, and also is the source of the official statistics on cause of death. The legal death certificate is the legal document required in order to proceed with the burial and any administrative procedure related to the deceased, such as legal proceedings related to inheritance, insurance, and pension payment, etc. Only an authorized judge of the Civil Registry can grant the legal death certificate. According to the General Law of Health, only physicians and personnel authorized by the MoH can fill out the medical death certificate. A hard copy of the medical death certificate is collected by the regional offices of INEGI and sent to their national headquarters where the automated coding to generate official figures takes place.

### Procedure

This study is part of the PHMRC project. Following the protocol of that study, once access to the medical records of each hospital was granted, we began a general review of the mortality database of every hospital in the study to identify potential gold standard cases. When a potential case was identified, a trained physician reviewed the medical record, and when available, the autopsy report, to classify the case as one of the three levels of gold standard: level 1, 2A, and 2B.

Review of medical records was carried out by six physicians who had received extensive training. A standardization and pilot study was carried out before the review of cases began. The physician team remained under strict supervision, and weekly meetings were held with all the members to review special cases and harmonize decision criteria.

As part of the PHMRC study to validate the verbal autopsy questionnaire, verbal autopsy interviews were conducted with relatives of deceased persons whose diagnoses were classified as gold standard. For the current study, identification numbers for the death certificates of successfully interviewed gold standard cases were recovered from the hospitals and provided to personnel of the General Directorate of Health Information of the MoH. They, in turn, provided us with the coding for the underlying and other causes of death stated in the medical death certificate. The research protocol of this study was approved by the ethics and research committee of the National Institute of Public Health and of the participant institutions that required it.

### Analysis

As it has been shown elsewhere, to assess concordance and accuracy between a diagnosis considered true (gold standard) and the underlying cause of death in official vital registration, it is necessary to use performance metrics that allow us to make comparisons with different cause-specific mortality fraction (CSMF) compositions and variable cause lists [17]. We conducted the analysis using a list of 27 causes for adults, seven causes for children, and five causes for neonates including stillbirths (Additional file 1).

Because we had information not only on the underlying cause of death, but also on the sequence of causes of death stated in the death certificate, we estimated concordance first considering only the underlying cause of death and then considering all causes of death recorded in the death certificate.

We calculated the chance-corrected concordance for each cause of death and the median chance-corrected concordance as a summary measure by age group for adults, children, and neonates. Chance-corrected concordance constitutes a measure of concordance between two classification methods (in this case the assignment of causes in the official figures and the gold standard), correcting for the probability of agreement expected by chance.

In addition, we calculated the median CSMF accuracy, which is a summary of the performance of a method in estimating CSMFs in the sample. Because results for both chance-corrected concordance and CSMF accuracy can be extremely sensitive to the CSMF composition of the test set, it is essential to report results for a sufficiently large set of randomly-generated CSMF test sets with different CSMF compositions. These CSMF compositions should be drawn randomly from an uninformative Dirichlet distribution [18]. To avoid bias, we used 500 test datasets (splits) from an uninformative Dirichlet distribution to estimate how well the estimated CSMFs compare to the true CSMFs, and we generated scatterplots to show the association between true and estimated CSMFs for each split. We also calculated a linear regression for each cause. The slope and intercept measure how accurately the estimated cause matches the true cause, with a slope of 1 and intercept of 0 indicating a perfect match. The root mean square error (RMSE) indicates how precisely the cause is estimated, with lower RMSE values indicating greater correlation.

## Results

### Individual cause assignment

For this study, a total of 8,573 medical records of deaths from 36 hospitals were reviewed. Gold standard levels were assigned to 2,995 cases and informed consent was granted by participants to apply the verbal autopsy in 2,031 of those cases for deaths that had occurred in 2009. It was possible to recover information on the medical death certificate of 1,729 deaths from 2009 included in the verbal autopsy database, and this information was sent to the General Directorate of Health Information to recover the underlying cause of death in official statistics for those deaths. Most of the cases for which we could not recover the information from the medical death certificate were violent deaths (for which the medical death certificate was not available at the hospital). Because we could not identify their underlying cause of death in the official statistics, 140 cases were dropped from the analysis (many of these were stillbirths or violent deaths). The final analysis included 1,589 deaths from 34 hospitals. The mean number of causes of death mentioned in each medical death certificate for generation of the underlying cause of death was 2.97 (95% uncertainty interval [UI]: 2.92, 3.02) for adults, 3.18 (3.00, 3.36) for children, and 2.40 (2.18, 2.61) for neonates.

The first step in the analysis was to assess the concordance between the cause of death that appeared in the medical death certificate and the gold standard for each age group, shown in Table 1. When analyzing only the underlying cause of death, the median chance-corrected concordance ranged from 38.5% for children to 66.5% for adults. When analyzing the concordance considering the sequence of causes of death mentioned in the medical death certificate compared to the gold standard, the median chance-corrected concordance increased, ranging from 58.9% for neonates to 75.9% for adults. This increase was the most substantial for children.

**Table 1:** Median chance-corrected concordance (%) by age group, for underlying diagnosis and all diagnoses

	Underlying Diagnosis		All Diagnoses	
	
	Median	95% UI	Median	95% UI
**Adults**	66.5	(65.9, 66.9)	75.9	(75.4, 76.3)

**Children**	38.5	(37.0, 40.0)	64.0	(61.4, 66.3)

**Neonates**	54.3	(52.2, 55.6)	58.9	(56.9, 60.5)

A detailed analysis of the concordance between underlying causes of death from the medical death certificate versus the gold standard is presented in Additional file 3. As we can see in this analysis, some causes, like diabetes, are more often recorded in the medical death certificate than in the gold standard, suggesting that the physicians are overstating this cause in the medical death certificates. There is also important misclassification of diarrhea, pneumonia, burns, lung cancer, falls, and poisonings. Other causes such as AIDS, cervical cancer, and leukemia/lymphomas have very little misclassification.

**Figure 1:**
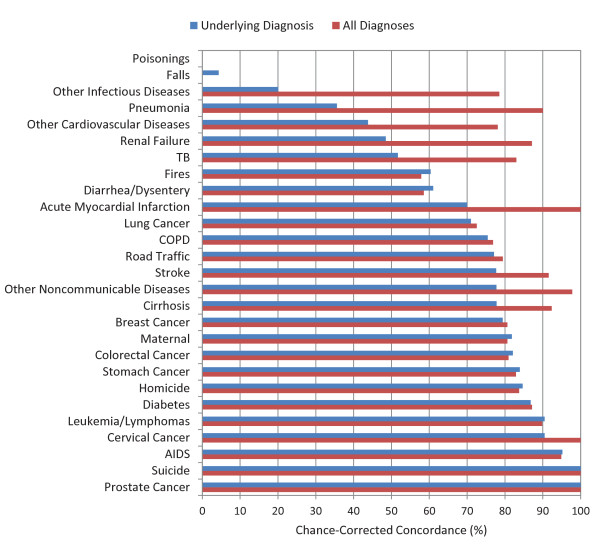
**Median chance-corrected concordance (%) by adult cause, for underlying diagnosis and all diagnoses**.

**Figure 2:**
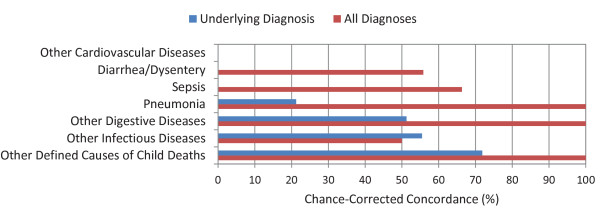
**Median chance-corrected concordance (%) by child cause, for underlying diagnosis and all diagnoses**.

**Figure 3:**
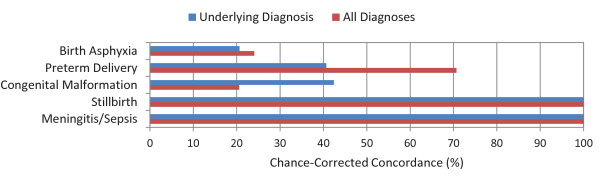
**Median chance-corrected concordance (%) by neonate cause, for underlying diagnosis and all diagnoses**.

The median chance-corrected concordance between the medical death certificate and the gold standard varied substantially by cause of death, shown in Figure 1 for adults, Figure 2 for children, and Figure 3 for neonates. For adults, prostate cancer, suicide, AIDS, leukemia/lymphomas, and cervical cancer had the highest concordances, while other infectious diseases, falls, and poisonings had a lower concordance. For children, the highest concordance was found for other infectious diseases and other defined causes and the lowest for other cardiovascular diseases. In the case of neonatal deaths, stillbirths and meningitis/sepsis had the highest concordance, and birth asphyxia had the lowest. Detailed values for chance-corrected concordance are given in Additional file 4.

### CSMF estimation

We estimated the CSMF accuracy of the medical death certificate in predicting the cause of death as identified in the gold standard, shown in Table 2. The accuracy indicates the ability of the medical death certificate to resemble the CSMFs as they are according to the gold standard. When considering only the underlying cause of death, the median accuracy ranged from 0.683 for child deaths to 0.780 for adults. Median accuracy increased when considering the mention of any cause of death in the medical death certificate versus the gold standard, ranging from 0.822 for child deaths to 0.887 for neonatal deaths.

**Table 2:** Median CSMF accuracy by age group, for underlying diagnosis and all diagnoses

	Underlying Diagnosis		All Diagnoses	
	
	Median	95% UI	Median	95% UI
**Adults**	0.780	(0.774, 0.785)	0.839	(0.835, 0.846)

**Children**	0.683	(0.663, 0.701)	0.822	(0.802, 0.845)

**Neonates**	0.756	(0.747, 0.769)	0.887	(0.880, 0.895)

**Figure 4:**
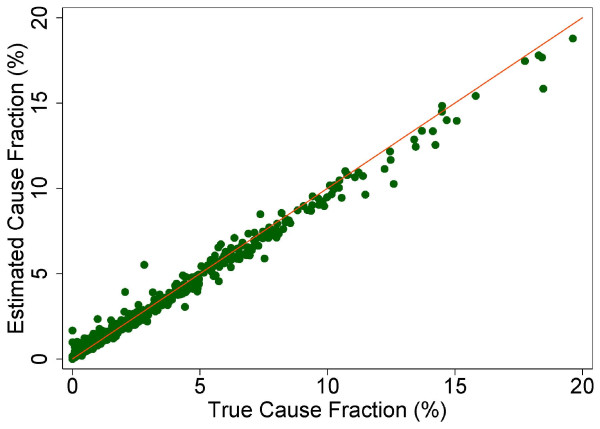
**Estimated versus true CSMFs across 500 Dirichlet splits for adult AIDS**.

**Figure 5:**
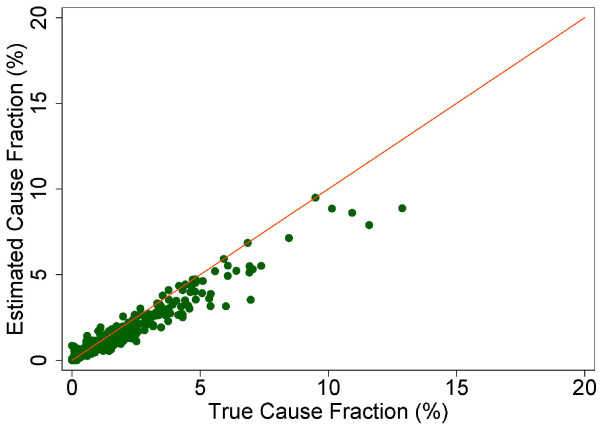
**Estimated versus true CSMFs across 500 Dirichlet splits for maternal deaths**.

**Figure 6:**
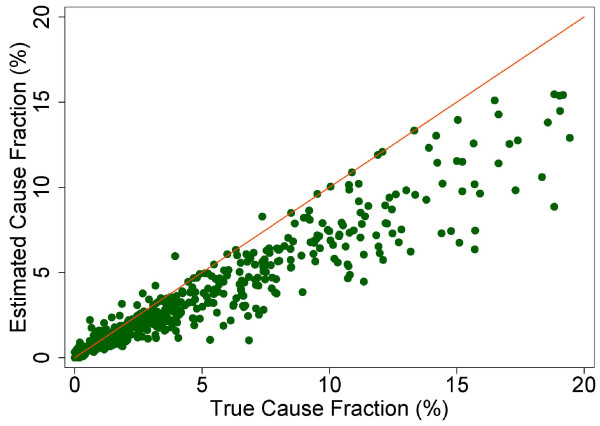
**Estimated versus true CSMFs across 500 Dirichlet splits for adult lung cancer**.

**Figure 7:**
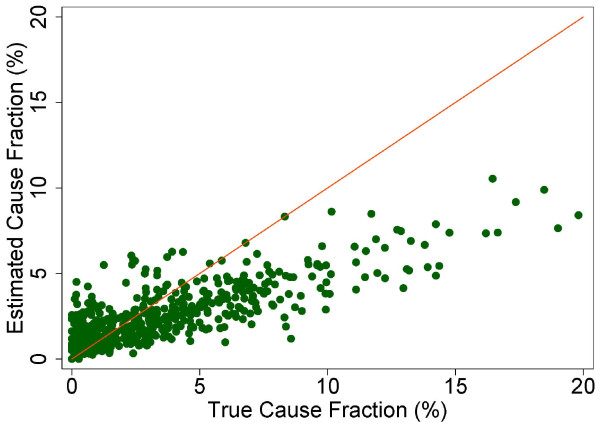
**Estimated versus true CSMFs across 500 Dirichlet splits for adult pneumonia**.

**Figure 8:**
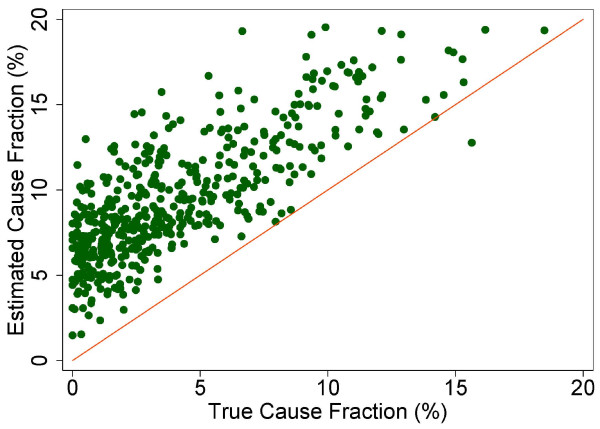
**Estimated versus true CSMFs across 500 Dirichlet splits for adult diabetes**.

**Figure 9:**
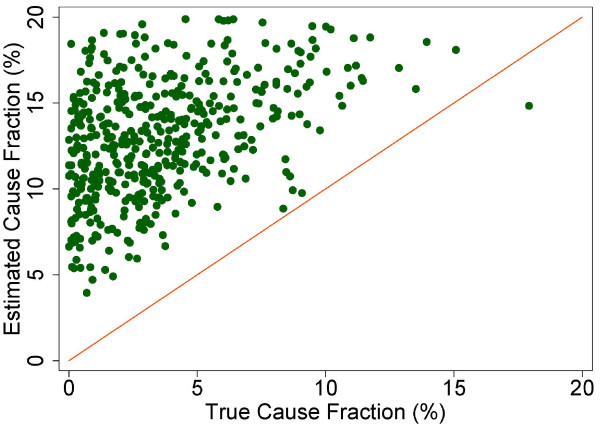
**Estimated versus true CSMFs across 500 Dirichlet splits for adult other noncommunicable diseases**.

The true and estimated CSMFs vary substantially across the 500 Dirichlet splits. Figures 4 through 9 show estimated versus true CSMFs for AIDS, maternal deaths, lung cancer, pneumonia, diabetes, and other noncommunicable diseases in adults. The red line indicates perfect concordance between estimated and true CSMFs, and data points closer to the red line more accurately predict the CSMF for a particular cause. As we can see, in the case of AIDS (Figure 4) the accuracy is very high for different true CSMFs. In the case of maternal deaths (Figure 5), the death certificate overestimates the occurrence of these deaths when the true CSMF is low but underestimates it when the true CSMF is higher. In the case of lung cancer (Figure 6), the death certificate overestimates when the true CSMF is very low, while it underestimates the occurrence of these deaths when the true CSMF increases. In the case of pneumonia (Figure 7), the accuracy is very low, overestimating at low levels of the true CSMF and underestimating for high true CSMFs. For diabetes (Figure 8) and other noncommunicable diseases (Figure 9), we see a substantial overestimation in the number of cases at any level of the true CSMF. Additional file 5 shows the slope, intercept, and RMSE results from the linear regression by cause. As expected, high-accuracy causes (AIDS) have a slope near 1 and intercept near 0, while low-accuracy causes (diabetes, other noncommunicable diseases) have a lower slope and higher intercept. Similarly, high-precision causes have a low RMSE and vice versa.

## Discussion

The importance of evaluating reliability and validity of underlying causes of death in mortality statistics has been recognized for a long time in the area of public health [19,20]. Generation of reliable statistical mortality data requires precise and consistent cause of death data, which in turn depends on the completeness and accuracy of cause of death diagnoses on the medical death certificate and its correct completion.

There are different approaches in assessing the accuracy of the diagnoses in the medical death certificates. For example, several publications have used postmortem results from autopsies as a gold standard to compare agreements and errors with medical death certificates. A meta-analysis of 53 autopsy series published in 2003 yielded a median error rate of 23.5% (range: 4.1%-49.8%). The analysis of diagnostic error rates in the same study, adjusting for the effects of case mix, country, and autopsy rate, yielded relative decreases of 19.4% (1.8%, 33.8%) for a period of 10 calendar years [21]. Not all diseases can be diagnosed with a postmortem examination. Adequate clinical examinations prior to death are also useful for correct determination and certification of causes of death. Using medical records as a gold standard (with review by pathologists or nosologists), some studies have validated the quality of death certificates in different countries. These include a population-based study of 1,068 deaths in Valencia, Spain [22] and another that was a review of 2,813 medical death certificates in Finland [23]. We calculated, for both studies, the same metrics that we used for our sample. In the first study the median chance-corrected concordance was 58.9% and in the second 60.3%. The accuracy was 0.94 and 0.90, respectively. It is important to mention that when we calculated the same metrics for only 1,284 adults we computed a mean chance-corrected concordance of 66% and a CSMF accuracy of 0.85 without sampling across the 500 Dirichlet splits.

To the best of our knowledge, this is the first study in Mexico assessing the validity of medical death certificates using a robust gold standard. Although the sample may be biased (more than 66% of the cases came from hospitals with high technical capabilities for diagnoses as well as good pathology departments) the results are consistent with other studies that used a sample of hospital deaths. Johansson and Westerling published a study of 31,785 death certificates that were linked to the national hospital discharge register and found an agreement of 46% with the main diagnosis of the hospital discharge and the underlying cause of death in medical death certificates [24]. For deaths that occurred in the hospital, the agreement increased to 84%, but for those that occurred at home, the agreement fell to 43%. The same study found an incremental trend of the agreement by age: 43.8% in children under 1 year old, 44.7% in children from 1 to 14 years of age, and 49% in adults aged 15 and over.

Our study found a reasonably high concordance and accuracy of the assignment of individual causes of death in the underlying cause of death of medical death certificates compared to the gold standard.

For adults, the list of 34 causes of death used in our study is reasonable and captures the epidemiological pattern for causes of death in the Federal District and Morelos, but this is not the case for the 21 causes for children. There were difficulties in obtaining the quota of deaths for some diseases, particularly for children aged 1 month to 12 years. According to official statistics, there were 868 deaths in the MoH health facilities of the Federal District and the state of Morelos in 2009. That year there were no deaths due to measles, meningitis, encephalitis, hemorrhagic fever, malaria, or bites of venomous animals in those age ranges, and there were only 39 deaths (4.5%) related to injuries, 28 in the Federal District and 11 in Morelos. None of these cases fit the inclusion criteria due to the lack of quality of the medical records. In the case of neonates we did not find any deaths due to pneumonia.

This study also shows a substantial variability in the concordance and accuracy depending on cause of death. In the case of adults, it is worthy to mention that for diabetes, a highly prevalent disease considered the number one cause of death in Mexico, this analysis shows a substantial overreporting of deaths based on the death certificate. Previous studies have shown that validity and comparability of diabetes can be affected because the diagnosis usually appears in only two-thirds of death certificates for people who had diabetes before death [25,26]. The order of the sequence of causes can also be a factor in whether or not diabetes is assigned as underlying cause of death [27,28]. Murray et al. show that when controlling for individual and community factors, mortality from diabetes can be reduced by 10% in the US and 24% in Mexico [29]. In this study we have seen a poor performance of diabetes CSMF prediction despite high chance-corrected concordance (86.8%) due to an overlap between diabetes in 38% of cardiovascular deaths and in 32% of pneumonia deaths.

It is clear, on the other hand, that chance-corrected concordance and CSMF prediction are good for diseases where the diagnosis should be evidence-based, such as HIV/AIDS, leukemia/lymphomas, and cervical cancer. More than 95% of the death certificates with these causes match the gold standard and have concordance over 90%. There are other causes, such as cirrhosis, homicides, and maternal deaths for which more than 95% of death certificates match the gold standard, but their chance-corrected concordance is lower than 85%. The case of maternal deaths is important to highlight because Mexico has undertaken a major effort since 2002 to improve the completeness and quality of their diagnoses. Chance-corrected concordance for maternal deaths was 80%, which included false positive cases diagnosed as HIV/AIDS and noncommunicable diseases that could have been considered indirect obstetric deaths.

The low concordance and accuracy in the case of child and neonatal deaths, as well as the variability across causes at these ages, could be associated with different factors, such as the type of causes selected as gold standard, the number of gold standard cases gathered by cause, and death certification itself. Regarding the last point, in Mexico, as in many other countries, death certification is perceived as unglamorous routine paperwork or a "burdensome task" of low priority. It is sometimes even interpreted as punishment or a task for doctors with a low level of training. This may be the case in the pediatric hospitals because the correlation between the medical death certificates and the gold standard was very low for all causes. In our study, when we considered not only the underlying cause of death, but the mention of any cause of death in the medical death certificate, the median chance-corrected concordance for children increased from 38.5% to 64.0% with a very dramatic increase in diarrhea, sepsis, and pneumonia. In neonates, the median chance-corrected concordance increased from 54.3% to 58.9%, mainly due to an increase in the concordance of birth asphyxia and preterm deaths. This is consistent with Hunt and Barr [30] who demonstrated in their study that including all causes written in the medical death certificates regardless of the sequence of diagnosis increased the concordance from 58% to 91% in neonatal deaths. In other words, the medical knowledge to assign a cause of death is present, but it could be used more efficiently in correctly filling out the death certificates.

These results suggest that using multiple cause of death analysis could better support decision-makers, because assigning "one cause to one death" is an exercise that is not easily understood by physicians, and this directly affects the reliability of the cause of death statistics. This problem becomes apparent when we consider all the causes reported on the medical death certificate, where the consistency of individual cause assignment and accuracy of the CSMF composition improve significantly. However, improving the quality of medical certification by using the multiple cause approach does not help to increase the validity of the cause of death statistics themselves because they are based on the underlying cause of death.

This study had various methodological strengths: in contrast to other validation studies using medical records as the gold standard, the cases selected in this study were based on robust gold standard criteria used in a multisite study; in addition, the metrics used to assess the performance of the VR system (chance-corrected concordance, CSMF accuracy, and linear regression, all estimated using a set of 500 test splits) are less sensitive to the cause composition of the test sample than other metrics traditionally used to assess performance, such as sensitivity and specificity.

The study had some limitations that should be considered in the interpretation of results. It is important to take into account that the cases included in this study are a sample of cases with complete medical records, which allowed their classification as gold standard. The cases came mostly from high-specialty hospitals in the Federal District and as a result may have better death certification than deaths taking place in nonspecialty medical units. For the same reason, the concordance and accuracy reported in this paper may be higher than one we might find in other settings. This study is based on high quality registries and cannot be extrapolated to the entire country.

It could be argued that the concordance may be affected not only by the information registered in the medical death certificate, but also by the coding procedures of the underlying cause of death. In this study, we used the coding information from INEGI, which generates the official mortality figures, and we assume that their procedures follow robust quality standards. However, the effect of possible coding problems on concordance and accuracy should be the subject of future research.

In addition, the sample size was small for child and neonatal deaths, which may have limited our ability to analyze concordance and accuracy in these age groups. The reduced sample size can be explained by the low mortality in these age groups in medical units of the Federal District, as well as by the presence of a different mortality pattern in the study area.

## Conclusions

Using a different approach to test the quality of the underlying cause of death for a sample of deaths from high-specialty hospitals, this study shows high concordance for some causes of death in adults but not for children and neonates. However, in future studies it would be worth including more causes of death in each category to reduce the size of the residual categories and better capture the epidemiological profile of a middle-income country. The results indicate the need to improve death certification procedures, especially in the case of children and neonates. While mortality in neonates and children under 12 has decreased significantly in recent years, it is desirable to improve the quality of records to better target health policies related to these age groups. Although we know our results are not generalizable to the rest of the country, it is important to consider that quality may be lower elsewhere. Each year there are an average of 40,000 deaths in these age groups (7% of total deaths), and in some states in Mexico the relative contribution reaches 10% or more. This requires an important commitment from the health system and health institutions and a review of coding procedures for deaths. It is necessary to provide tools and training to physicians so they can conduct a proper certification of the deaths. Evidence in the literature suggests that this is feasible [31,32], and manuals exist that can assist in putting this into practice [33]. It is crucial to address the issue of the importance of an accurate certification of cause of death and to implement quality controls in medical institutions. In terms of research, this study underlines the need to expand analysis of this type to other areas of the country using similar robust gold standards.

## Abbreviations

ACME: Automated Coding of Medical Entities; CEMECE: Center for Disease Classification in Mexico; CSMF: cause-specific mortality fraction; INEGI: National Institute of Statistics and Geography, Mexico; MoH: Ministry of Health; PHMRC: Population Health Metrics Research Consortium; VR: vital registration.

## Competing interests

The author declares that they have no competing interests.

## Authors' contributions

BH participated in the design of the project and the acquisition, analysis, and interpretation of data. He wrote the first draft. DRV, MR, and SG participated in the acquisition and analysis of data. CA contributed in the analysis of data, and RL is the principal investigator of the project and participated in the conceptualization, design, data collection, analysis, and interpretation. He revised the first draft and wrote the final version. All authors read and approved the manuscript.

## Supplementary Material

Additional file 1 Number of deaths for adults, children, and neonates by cause of death Click here for file

Additional file 2 List of participant hospitals and number of cases obtained from each hospital Click here for file

Additional file 3 Misclassification matrix showing causes from the gold standard and medical death certificate Click here for file

Additional file 4 Median chance-corrected concordance by age group and cause, for underlying diagnosis and all diagnoses Click here for file

Additional file 5 Slope, intercept, and RMSE from linear regression of estimated versus true CSMFs, by age group and cause Click here for file
